# The Unconventional Viruses of Ichneumonid Parasitoid Wasps

**DOI:** 10.3390/v12101170

**Published:** 2020-10-15

**Authors:** Anne-Nathalie Volkoff, Michel Cusson

**Affiliations:** 1 Diversity, Genomes, Insects-Microorganisms Interactions (DGIMI), Université de Montpellier, Institut National de Recherche pour L’Agriculture, L’Alimentation et L’Environnement (INRAE), 34095 Montpellier, France; 2 Laurentian Forestry Centre, Natural Resources Canada, Quebec City, QC G1V 4C7, Canada; michel.cusson@canada.ca

**Keywords:** parasitoid wasp, ichnovirus, virus-like particles, endogenous viruses, mutualistic viruses, genomic architecture

## Abstract

To ensure their own immature development as parasites, ichneumonid parasitoid wasps use endogenous viruses that they acquired through ancient events of viral genome integration. Thousands of species from the campoplegine and banchine wasp subfamilies rely, for their survival, on their association with these viruses, hijacked from a yet undetermined viral taxon. Here, we give an update of recent findings on the nature of the viral genes retained from the progenitor viruses and how they are organized in the wasp genome.

## 1. Introduction

**Figure 1:**
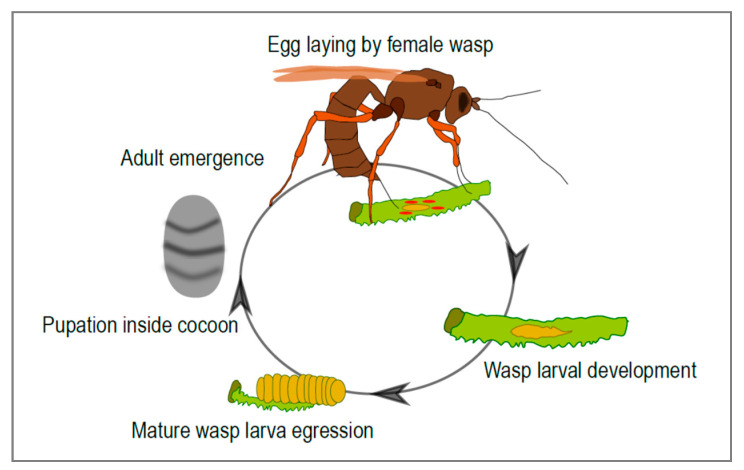
Life cycle of an ichneumonid parasitoid wasp associated with mutualistic viruses. The adult female wasp injects virus particles along with the eggs during parasitization. The progeny develops within the body of the parasitized insect. At the end of larval development, the mature larva egresses from the host and spins a cocoon where pupation takes place. A free-living adult then emerges from the cocoon.

The ichneumonid wasps (Hymenoptera: Ichneumonidae) form a very large and diverse group of insects [1] that share a life cycle involving parasitism. Around 25,000 ichneumonid species have been described to date [2], but their actual numbers are estimated to be about four times this high [3]. Many ichneumonid species are endoparasitoids: the females lay their eggs inside another insect, usually an insect larva, where their progeny develops (Figure 1). Amazingly, in many cases the parasitized insect continues its development, and usually only dies once the parasitoid has completed its larval development. This life history strategy presents a number of challenges for the parasitoid. In particular, the developing wasp needs to escape the immune response of its host and modulate host development and metabolism in a way that is beneficial to its own development. There are many strategies parasitoids employ to inhibit host immune defenses and to finely tune host development to suit their own. The females of some species, for instance, produce specific immunosuppressive proteins in their venom gland and inject them into the host during parasitization. Another amazing strategy, described only in some ichneumonid and braconid subfamilies, involves the production, in the female genital tract, of virus-like particles that allow the transfer of effector molecules from the wasp to the host. Strikingly, these entities were recently discovered to result from the “domestication” of conventional viruses in these wasp lineages.

## 2. A Brief History About the Discovery of Mutualistic Viruses in Parasitoids

Virus-like particles associated with parasitoids and necessary for successful parasitism were discovered in the 1970s, the first example being the immunosuppressive particles described in the ichneumonid wasp *Venturia canescens*, a parasitoid of pyralid caterpillars [4]. Subsequent studies showed that these particles, produced in the wasp ovaries, were composed of wasp proteins and devoid of nucleic acids [5]. Concomitantly, other particles resembling viruses were described in various wasp species belonging to the families Ichneumonidae and Braconidae [6,7]. In contrast to those found in *V. canescens*, these particles contained multiple circular, double-stranded DNAs, with aggregate genome sizes ranging from 150 to more than 600 kb [8,9]. Like the particles of *V. canescens,* they are produced in wasp ovaries and injected into the host along with the parasitoid egg. Particles then rapidly infect the parasitized host’s cells, which leads to expression of viral genes [10] and alterations of host physiology necessary for the successful development of the wasp progeny [11,12]. Early work also highlighted some atypical features of these viruses: although they contain DNA, they do not replicate in the parasitized insect [13] and are only transmitted vertically through the wasp germline [14]. It was further shown that the DNA segments included in these particles are indeed maintained integrated in the wasp genome [15]. However, and despite their atypical life cycle, they were formally recognized in 1991 by the International Committee on Taxonomy of Viruses as members of a family of insect viruses, named *Polydnaviridae* [16], although their status as true viruses has been, and remains, a contentious issue.

The polydnavirus (PDV) family includes two taxa, the bracoviruses (BVs) and the ichnoviruses (IVs), associated with wasps from the braconid and ichneumonid families, respectively. Although they share a common life cycle, BVs and IVs were separated in two groups because of their morphological differences (BVs have cylindrical nucleocapsids of variable length surrounded by a single envelope; IVs have lenticular nucleocapsids more or less uniform in size, enveloped with two membranes). Subsequent studies confirmed their distinctive features: they differ in the nature of the genes harbored by their packaged genomes [17] and derive from distinct viral ancestors [18,19].

Virus-like particles devoid of nucleic acids have recently been described in the ovaries of the braconid wasp *Fopius arisanus* [20]. We may well be only at the beginning of discoveries of viruses and virus-like particles associated with, and used by, parasitoid wasps.

## 3. Diversity of Ichneumonid Virus Particles

Virus particles associated with ichneumonid wasps have so far been described only in two ichneumonid subfamilies, the Campopleginae and the Banchinae. However, surveys have been made essentially by electron microscopy, a technique that does not permit screening of large numbers of wasps [21]. With the recent development of new sequencing technologies, new viruses will likely be discovered in the near future.

**Figure 2:**
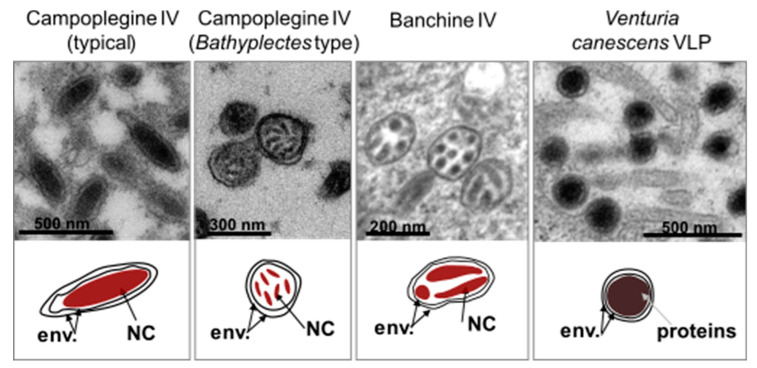
Morphological differences between virus particles found in ichneumonid wasps. Upper panel. Transmission electron micrographs of female oviduct transversal sections; particles shown are observed in the calyx lumen for campoplegine IVs (typical and *Bathyplectes* types) and *Venturia canescens* VLPs; particles are shown in the nucleus of calyx cells for banchine IVs. Lower panel. Schematic representation of the secreted particles. cyt, cytoplasm; env., particle envelopes, NC, nucleocapsid.

Campoplegine and banchine wasp species both harbor polydnaviruses of the ichnovirus group. Banchine IV virions typically harbor several nucleocapsids [22,23] whereas most virions of campoplegine IVs contain a single nucleocapsid (Figure 2). However, virions with multiple nucleocapsids have recently been described for the campoplegine wasp *Bathyplectes* sp., which parasitizes coleopteran larvae [24]; the other campoplegine wasps that have been shown to carry IVs develop in lepidopteran larvae. Banchine and campoplegine IVs also differ with respect to their packaged genomes: the former have far more segments than the latter, and the genes they contain are strikingly different (Table 1).

To date, *V. canescens* is the only campoplegine species known to produce particles that do not contain DNA. Whether other species from this lineage also produce VLPs instead of typical ichnoviruses is unknown.

## 4. Evidence for a Viral Origin of Ichnoviruses

Sequencing of PDV packaged genomes revealed the absence of typical virus replication genes in the DNA transferred to the parasitoid’s host. This finding was in agreement with previous work showing that PDVs are not replicative in the parasitized insect. The packaged genome encodes hundreds of genes (Table 1). Most are expressed in the parasitized insect and responsible for the physiological alterations observed in the parasitized host. Many of them have similarities with eukaryotic genes, suggesting that most of these genes have an insect, probably a wasp, origin rather than a viral origin [27].

The above observation raised questions about the viral nature of PDVs: were they real viruses, or just virus-like wasp secretions? To address this question, an idea emerged in the scientific community: to search for virion structural proteins via proteomic analysis of purified PDV particles. Until then, only two PDV structural proteins had been characterized in the campoplegine wasp *Campoletis sonorensis* and shown to correspond to genes that are not encapsidated [28,29].

### 4.1. The Virus Ancestor and the Virus-Derived Machinery Responsible for the Production of Campoplegine IV Particles

An analysis of the transcriptome associated with the wasp replicative tissue (calyx), coupled with mass spectrometry analysis of purified particles, led to the identification of ~40 genes involved in *Hyposoter didymator* ichnovirus (HdIV) particle production [19]. These genes included homologues of the IVp12 and IVp53 genes originally identified in *C. sonorensis* [28,29]. All were specifically expressed in the calyx of the wasp and at least 19 of them encoded components of virus particles. It was also shown that these genes are not packaged in the particles, but reside permanently in the wasp genome. However, no clear sequence similarity with genes from known viruses was detected.

Furthermore, homologs to HdIV structural genes were identified in the calyx transcriptome of other IV-associated campoplegine wasps, namely *Tranosema rostrale* [19] and *Bathyplectes* sp. [24], or in the genome of *C. sonorensis* [30]. This clearly indicated that the set of virion structural genes was conserved among campoplegine wasps known to produce IVs.

**Figure 3:**
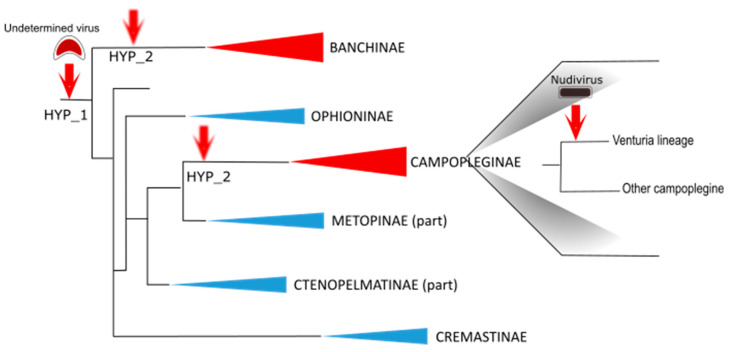
Schematic diagram illustrating virus acquisitions during ichneumonid evolution. A virus of undetermined nature was captured by an ichneumonid ancestor. It is not known whether the same event of integration (hypothesis HYP-1) or two distinct events with related viral progenitors (hypothesis HYP-2) led to the ichnoviruses presently associated with campoplegine and banchine. Remarkably, virus particles were described in the Campopleginae and Banchinae (indicated in red), but never in other ichneumonid subfamilies (indicated in blue). Subsequently, another event of virus integration occurred in the *Venturia* lineage of the campoplegine subfamily: an alphanudivirus was captured, giving rise to the virus-like particles described in the species *Venturia canescens*. Schematic phylogenic tree drawn based on Bennett et al., 2019 [1].

The genes involved in IV particle formation are clustered in specialized regions of the wasp genome that were named “Ichnovirus Structural Protein Encoding Regions” (IVSPERs). IVSPERs display features that make them distinct from the rest of the wasp genome, including a high gene density relative to regions of the wasp genome containing cellular genes, and genes that are all made of a single exon, which is more typical of conventional virus genes than of wasp genes, generally consisting of multiple exons. Therefore, despite their lack of similarity with known viral genes, the organization and content of IVSPERs provided evidence that they derived from a viral ancestor whose genome had been integrated and retained in the wasp genome (Figure 3).

The recent sequencing of the *H. didymator* and *C. sonorensis* genomes [30] led to the identification of two additional IVSPERs and one isolated structural gene in *H. didymator*, and of four IVSPERs and two singletons in *C. sonorensis*, adding up to 54 and 48 genes, respectively, in the virus-derived machineries of these two wasp species.

### 4.2. A Campoplegine-Related Virus-Derived Machinery Involved in Banchine IV Production

A search for virion components was then undertaken for IVs associated with species from the other ichneumonid subfamily, the Banchinae, which produces particles that differ in both their morphology and gene content, compared to campoplegine wasps. The analysis was conducted for the species *Glypta fumiferanae* using the same approach [31].

Strikingly, the structural genes that were identified displayed strong similarities with those identified in campoplegine wasps and were similarly clustered in IVSPERs. A total of almost 60 genes were identified, distributed among three IVSPERs, including 26 genes showing strong similarity with *H. didymator* IVSPER genes. These results provided clear evidence that the banchine and campoplegine IV ancestors were related. Whether the two types of IVs derive from a single virus integration event or from two distinct events of acquisition of related viral ancestors remains unknown (Figure 3).

Interestingly, *G. fumiferanae* IVSPERs also contained at least three genes with conserved viral domains, namely a DNA polymerase, a D5 primase-helicase, and a DEXD helicase. Note that homologs for the last two genes were also recently described in *H. didymator* and *C. sonorensis* IVSPERs [30]. These domains are found in a set of genes conserved among nucleocytoplasmic large DNA viruses (NCLDVs), an observation that suggests the IV progenitor(s) could be from an uncharacterized or extinct viral taxon related to this group of viruses.

### 4.3. The case of Venturia canescens: An Example of Viral Symbiont Replacement

The virus particles produced in the campoplegine *V. canescens* are devoid of DNA. However, like IVs, they are produced in the nuclei of the wasp calyx cells and their morphogenesis resembles that of campoplegine IVs. Therefore, it was first thought that they corresponded to “defective” IV particles.

To explore the nature of these VLPs, the same approach described above was employed (calyx transcriptome and VLP proteomic analyses). This work revealed the presence of highly transcribed sequences displaying strong similarities with alphanudivirus genes. Nudiviruses are a sister group of baculoviruses, a well-known family of pathogenic insect viruses with which they share a set of core genes. A total of 51 nudiviral genes were identified in the *V. canescens* genome, organized in six clusters [32]. All nudiviral genes are specifically transcribed in the calyx cells, and ~25 of them encode proteins detected by mass spectrometry analysis of the VLPs. As with IVSPERs, nudiviral regions are amplified in the calyx cells when VLP production takes place. Altogether, these findings show that VLPs are produced by a nudivirus-derived machinery that originates from the integration of a nudivirus genome in the *V. canescens* lineage (Figure 3).

Based on the functions of their homologs in baculoviruses, nudiviral genes considered to be involved in the regulation of viral transcription (e.g., the RNA polymerase subunits lef-4, lef-5, lef-8, lef-9 and p47) and the production of viral envelope components (e.g., pif-0 to pif-6) were observed to have been retained by the *V. canescens* genome over the course of evolution and were expressed in calyx cells [32]. By contrast, core genes involved in viral DNA replication (e.g., DNA polymerase) or genes involved in nucleocapsid assembly or DNA packaging (e.g., 38K, vp39, vlf-1) were detected as pseudogenes and consequently were no longer functional [33]. Loss of the latter is consistent with the absence of packaged DNA in the VLPs.

Based on the phylogenetic tree that places *V. canescens* close to IV-associated species [34], the ancestor of *V. canescens* was expected to carry an IV. Indeed, an exploration of the wasp genome led to the identification of remnants of IV sequences, indicating that, in this wasp lineage, a second acquisition of a virus genome led to the functional replacement of the IV machinery by a nudiviral machinery, probably because of the selective advantage that producing VLPs (instead of IVs) conferred to the wasp.

Note that the VLPs of the braconid *Fopius arisanus* are similarly produced by a nudiviral machinery derived from the integration of an alphanudivirus [20]. Interestingly, braconid PDVs, the bracoviruses, also derive from the integration of a nudivirus, but in this case, the progenitor is a betanudivirus. Thus, in different parasitoid lineages, integration of related insect viruses (here, the nudiviruses) can lead to the evolution of different strategies enabling the wasp to develop inside its host, with each strategy being dependent on the genes selected and maintained in the wasp genome. In some cases, integration resulted in a machinery allowing the production of DNA-laden particles (i.e., the bracoviruses), while in others, the machinery evolved to enable the production of particles devoid of DNA and used strictly for the transfer of proteins to the host (i.e., the VLPs found in *V. canescens* and *F. arisanus*).

## 5. Genomic Organization of Ichnoviruses

**Figure 4:**
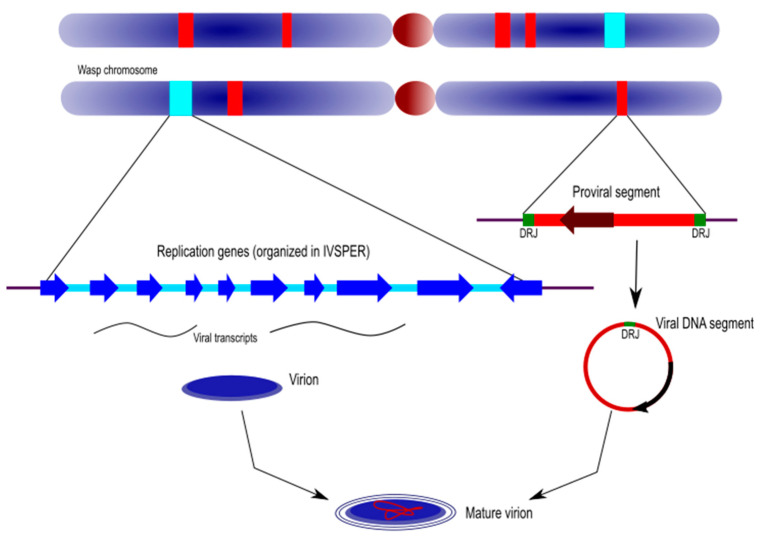
Genomic architecture of ichnoviruses. Ichnovirus loci are dispersed within the wasp chromosomes and comprise the proviral genome segments and the clusters of replication genes (or IVSPERs, for IV structural proteins encoding regions). The first are the linear templates of the circular molecules that are packaged in IV particles. They are flanked at each side by direct repeated sequences (named DRJ for “direct repeat junction”) allowing circularization of the molecule. The replication genes are transcribed in the wasp replicative tissue (calyx) and are involved in the production of the particle. See text for more details.

The recent whole genome sequencing of the two campoplegine wasps, *H. didymator* and *C. sonorensis*, revealed an extremely complex ichnovirus genomic architecture [30]. The integrated ichnovirus genome comprises two components that differ in both their features and functions in the virus/parasitoid life cycle: the proviral genome segments and the “replication” genes (Figure 4).

### 5.1. Ichnovirus Proviral Genome Segments

Proviral genome segments are here defined as the linear copies, within the wasp genome, of the circular genome segments packaged in IV particles. Proviral segments are characterized by direct repeats located at each end of their sequence. Only one copy of the repeat is detected in the circularized molecules, indicating that these repeated sequences (also named “direct repeat junctions”, or DRJs) correspond to excision sites where homologous recombination takes place.

The genomes of *H. didymator* and *C. sonorensis* both included a high number of proviral loci, with 54 identified in the former and 33 in the latter, most of which corresponding to a single proviral segment. Proviral segments were also extremely dispersed within the wasp genomes. When several viral loci were identified in a single wasp scaffold, most of the time they were separated by long portions of wasp genome (median size of 115.1 kb). This great dispersion was also confirmed by fluorescent *in situ* hybridization, which revealed the location of viral segments on different *H. didymator* chromosomes. In comparing the positions and genomic environments of proviral segments in *H. didymator* and *C. sonorensis*, the segments were observed to be located in genomic regions that differed between the two species. 

Comparison of the two species also highlighted divergence at the level of their DNA segments. *C. sonorensis* ichnovirus (CsIV) segments were on average longer (i.e., 6 to 23 kb) than those of *H. didymator* ichnovirus (HdIV) (i.e., 2 to 18 kb) and they contained a total of 111 predicted genes, as compared to 152 for HdIV. Although both CsIV and HdIV segments contained members of five ichnovirus-conserved multimember families (*repeat-element genes*, *vankyrins*, *vinnexins*, *cys-motif* and *N-genes*), they were in variable numbers in each species, and each genome harbored a number of IV genes specific to either *C. sonorensis* or *H. didymator*. 

Therefore, overall, proviral segments have gene contents and genomic locations that appear specific to a given wasp species, suggesting their contemporary features have been shaped by evolution in response to selective pressures favoring those that facilitate development of the immature wasp in its insect host.

### 5.2. Ichnovirus Replication Genes

Replication genes are those that descend from the viral ancestor; they are involved in virion production and are clustered in the wasp’s IVSPERs (see above).

**Figure 5:**
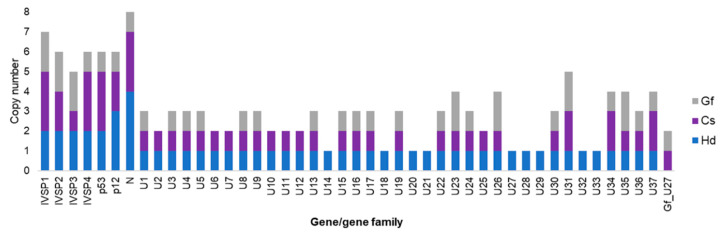
IVSPER gene conservation across ichneumonids. The numbers are for the copies of each gene or gene family identified in three ichneumonid species, Cs, *Campoletis sonorensis* (campoplegine); Gf, *Glypta fumiferanae* (banchine); Hd, *Hyposoter didymator* (campoplegine).

Globally, IVSPERs are far less numerous than proviral segments [30]. The *H. didymator* genome contained one isolated IV replication gene and five IVSPERs (with sizes varying from 2 to 27 kb and a number of genes ranging from 2 to 19), adding up to 54 predicted replication genes. Similarly, the *C. sonorensis* genome was observed to harbor 48 IV replication genes, including two isolated genes and 46 genes clustered in five IVSPERS (varying in size from 9 to 33 kb and containing from 3 to 19 genes). Regarding the nature of the replication genes, the majority (~80%) were shared by *H. didymator* and *C. campoletis* (Figure 5). Note that some genes were present in duplicates or triplicates, with the number of copies differing in some cases between the two wasp species. Interestingly, most of these genes are also conserved in the banchine species *G. fumiferanae* (Figure 5).

When the *H. didymator* and *C. sonorensis* IVSPERs are compared, we observe a high degree of conservation in terms of gene content and gene order (synteny). Finally, although only two campoplegine wasp genomes are currently available and both remain quite fragmented, comparison of the genomic regions containing IVSPERs revealed that two small clusters of replication genes were located in the same wasp genomic environment, suggesting a partial conservation of IVSPER location within the wasp genome.

Therefore, by contrast with genome segments, the replication genes, which are required to produce virus particles, are highly conserved between the two campoplegine wasps. Note that conservation of the viral machinery versus diversification of the viral segments is also observed in bracoviruses [35].

## 6. Morphogenesis of Ichnoviruses: Which Genes Are Involved?

A total of 54 genes potentially belonging to the ichnoviral machinery have been identified in the genome of *H. didymator*. Unfortunately, with rare exceptions, these genes do not show any similarity to known genes, and their functions in the process of viral particle production have yet to be elucidated. 

**Figure 6:**
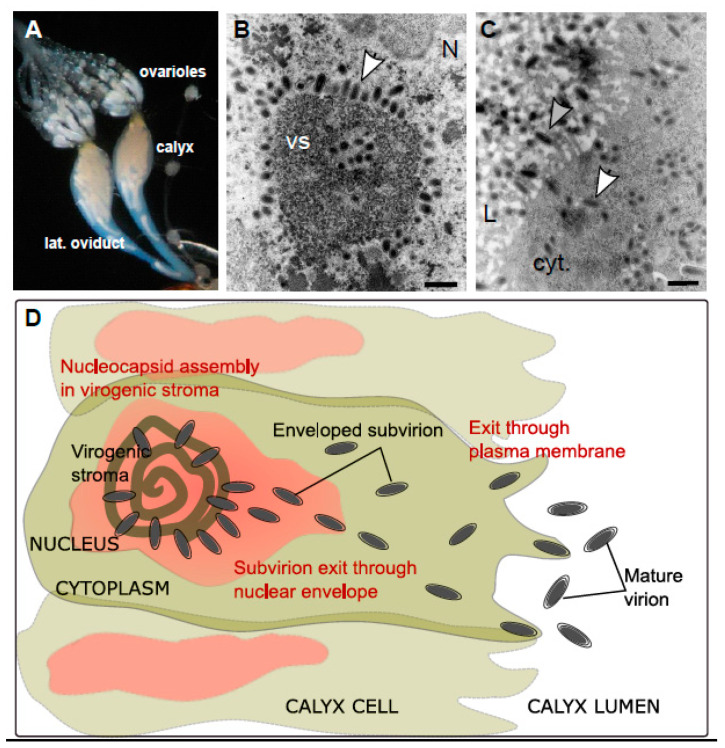
Ichnovirus morphogenesis. (**A**) Light microscopy view of the ovaries of a campoplegine wasp. The calyx region corresponds to the apical part of the lateral oviducts, just below the ovarioles where oogenesis takes place. (**B**) Transmission electron micrograph showing details of a calyx cell nucleus (N). Enveloped virions (arrowhead) are being assembled at the vicinity of the virogenic stroma (VS). Scale bar 500 nm. (**C**) Detail of the cytoplasm (cyt.) of a calyx cell presenting numerous virions (white arrowhead) that bud at the plasma membrane (PM) and accumulate as mature virions surrounded by two envelopes (grey arrowhead) in the calyx lumen (L). Scale bar 500 nm. (**D**) Schematic representation of the different steps of IV particle production in the wasp calyx cells.

Ichnoviruses have an atypical morphogenesis, with calyx cells being able to produce particles throughout the life of the female parasitoid. Virions surrounded by an envelope are assembled in the nucleus of calyx cells, after which they bud at the nuclear envelope, migrate to the plasma membrane and are secreted towards the lumen of the oviducts, acquiring a second envelope in the process (Figure 6).

A first functional study has been undertaken recently for six candidate replication genes, chosen because of their high level of expression in the replicative tissue (calyx) and because they encode proteins identified during the proteomic analysis of the purified viral particles [36]. Their involvement in particle formation was demonstrated through inhibition of their expression by RNA interference (RNAi), an approach that has proven very effective in this biological system: IVp12-1 is involved in the formation of the virogenic stroma, two genes (U23 and IVSP4-1) are involved in the assembly of nucleocapsids, two genes (U22 and IVSP3-1) are involved in the release of virions from the nucleus and IVp53-2 is involved in the release of virions from the cell.

This very first functional analysis of the production of particles of viral origin in an ichneumonid wasp clearly shows that the genes examined are necessary for morphogenesis and cell trafficking of viral particles, and that their functions are those expected of typical viral genes.

## 7. Concluding Remarks and Open Questions for Future Research

To our knowledge, parasitoids are unique in their capacity to domesticate viruses for their own benefit. Examples of endogenous viral elements maintained in eukaryotic hosts are numerous, but in most cases, these examples concern one or a few viral genes [37,38]. Here, a whole viral machinery was maintained in parasitoid genomes. This machinery allows the wasp to manufacture a delivery system that enables the transfer of effector molecules (proteins or genes) from the wasp to the insect in which the wasp progeny will develop.

From the discovery of these peculiar viruses until now, major advances have been made, particularly thanks to the new “omics” technologies. Nonetheless, there remain numerous unresolved questions regarding their evolutionary trajectories or the mechanisms underlying their production.

For instance, some aspects of IV replication have yet to be elucidated. For example, the functions of the remaining replication genes need to be assessed. More specifically, it is still uncertain whether some of these genes are involved in DNA amplification or whether this step relies entirely on the wasp cellular machinery.

The functions of the three NCLDV-like genes identified in the IVSPERs of *G. fumiferanae* [31], two of which were later found to have homologs in campoplegine wasps [30], should be investigated, perhaps using an RNAi approach similar to the one described above [36]. These genes were found to be overexpressed in the replicative calyx tissue of *G. fumiferanae*, relative to the ovarioles [31], and were hypothesized to be part of the replicative machinery of the progenitor virus. If the latter hypothesis is correct, whether these genes have conserved their original functions is an open question. The apparent loss of one of them in campoplegine wasps suggests that one or all of them are not essential for IV replication, although the possibility remains that they have retained a function in banchine wasps, while having lost it in their campoplegine couterparts. Should this be the case, it would point to different degrees of virus domestication achieved in these two groups of wasps. 

Regarding banchine IVs, virtually nothing is known about the arrangement/organization of the linearized genome segments within the wasp genome: are the segments isolated as in campoplegine wasps or clustered as shown for BV segments in braconid wasps? Answering this question will require that the genome of some IV-carrying banchine wasps be sequenced and assembled. The resulting data may shed light on the similarity or divergence of the evolutionary trajectories taken by campoplegine and banchine IVs. 

The observed dispersion of the IV segments within the wasp genome suggests that they can easily move within the host genome, somewhat like transposons, despite the fact that no integrase has been identified yet. Comparison of the degree of genomic dispersion of viral genome segments across many species of campoplegine wasps (only two species have been examined to date) may provide clues as to how their genomic architecture evolved. Interestingly, the fact that genome segments are typically isolated suggests that the mechanism underlying viral DNA amplification, prior to segment excision and encapsidation, does not necessitate their clustering in a given region of the wasp genome. Elucidation of how this amplification is coordinated across these isolated genomic pockets should be a rewarding endeavor. 

Along the same line, we may ask why genome segments tend not to be clustered in ichneumonid genomes whereas they are clustered in braconid genomes. In both cases, linearized segments are bordered, at each end, with direct repeats allowing homologous recombination and segment excision. However, whereas consensus sequences corresponding to excision sites are found in all BV DRJs, no conserved stretches of nucleotides have been identified in IV DRJs. It is possible that the different genomic organization of linearized segments and the different DRJ structures reflect different regulatory mechanisms for DNA molecule excision in the two types of PDVs. BV segments are amplified in replication units encompassing several segments. For IV, the limits of the replication units need to be investigated in the near future to get insights into the mechanisms allowing IV segment excision.

We propose here some avenues of research, but surely there are many other questions that will need to be addressed in order to better understand these highly unusual virus–parasitoid associations and how the virus particles are produced in ichneumonid wasps. In the future, this knowledge may also enable advances in virology and lead to applications in various fields, including medicine and agriculture.

## Figures and Tables

**Table 1:** Features of the sequenced packaged ichnovirus genomes from campoplegine and banchine ichneumonid species.

Parasitoid Species	PDV Name	Number of Segments and Size Range	Number of Predicted Genes	Nature of Most Abundant Gene Families	Reference
***Campopleginae***					
*Campoletis sonorensis*	CsIV	24 (6.1–19.6 kb)	101	*Viral ankyrins* *Repeat-element genes* *Cys-motif proteins* *Viral innexins* *N-genes*	Webb et al., 2006 [17]
*Hyposoter didymator*	HdIV	50 (2.5–3.6 kb)	135		Dorémus et al., 2014 [25]
*Hyposoter fugitivus*	HfIV	56 (2.6–8.9 kb)	150		Tanaka et al., 2007 [26]
*Tranosema rostrale*	TrIV	>40 (4.1–10.1 kb)	>89		Tanaka et al., 2007 [26]
***Banchinae***					
*Glypta fumiferanae*	GfIV	105 (1.5–5.2 kb)	101	*Viral ankyrins* *Protein tyrosine phosphatases (PTPs)* *NTPase-like proteins*	Lapointe et al., 2007 [22]
*Apophua simplicipes*	AsIV	>132 (~1.0–4.0 kb)	186		Djoumad et al., 2013 [23]
